# Supplementary material for: Pyruvate Dehydrogenase Kinase 1 inhibition mediated oxidative phosphorylation enhancement in cartilage promotes osteoarthritis progression
Source: BMC Musculoskelet Disord. 2023 Jul 20;24:597. doi: 10.1186/s12891-023-06585-6 (PMC10357736; doi:10.1186/s12891-023-06585-6)
Supplement: Supplementary file 1 — Additional file 1. [file 12891_2023_6585_MOESM1_ESM.zip › MF.Gsea.1653627316242/GOMF_MRNA_3_UTR_AU_RICH_REGION_BINDING.html]

Details for gene set GOMF\_MRNA\_3\_UTR\_AU\_RICH\_REGION\_BINDING[GSEA]

|  || Dataset | 55457\_collapsed\_to\_symbols.cls.cls #OA\_versus\_normal.cls.cls #OA\_versus\_normal\_repos |
| Phenotype | cls.cls#OA\_versus\_normal\_repos |
| Upregulated in class | normal |
| GeneSet | GOMF\_MRNA\_3\_UTR\_AU\_RICH\_REGION\_BINDING |
| Enrichment Score (ES) | -0.54293567 |
| Normalized Enrichment Score (NES) | -1.75239 |
| Nominal p-value | 0.0034482758 |
| FDR q-value | 0.07731533 |
| FWER p-Value | 0.741 |
Table: GSEA Results Summary

  

Fig 1: Enrichment plot: GOMF\_MRNA\_3\_UTR\_AU\_RICH\_REGION\_BINDING      
 Profile of the Running ES Score & Positions of GeneSet Members on the Rank Ordered List

  

| SYMBOL | TITLE | RANK IN GENE LIST | RANK METRIC SCORE | RUNNING ES | CORE ENRICHMENT || 1 | NUDT21 | nudix hydrolase 21 [Source:HGNC Symbol;Acc:HGNC:13870] | 1734 | 0.393 | -0.0838 | No |
| 2 | APOBEC1 | apolipoprotein B mRNA editing enzyme catalytic subunit 1 [Source:HGNC Symbol;Acc:HGNC:604] | 2695 | 0.280 | -0.1222 | No |
| 3 | HNRNPD | heterogeneous nuclear ribonucleoprotein D [Source:HGNC Symbol;Acc:HGNC:5036] | 4830 | 0.085 | -0.2769 | No |
| 4 | HNRNPA0 | heterogeneous nuclear ribonucleoprotein A0 [Source:HGNC Symbol;Acc:HGNC:5030] | 5485 | 0.033 | -0.3234 | No |
| 5 | EXOSC4 | exosome component 4 [Source:HGNC Symbol;Acc:HGNC:18189] | 5693 | 0.016 | -0.3373 | No |
| 6 | CPEB1 | cytoplasmic polyadenylation element binding protein 1 [Source:HGNC Symbol;Acc:HGNC:21744] | 6435 | -0.038 | -0.3900 | No |
| 7 | CPSF1 | cleavage and polyadenylation specific factor 1 [Source:HGNC Symbol;Acc:HGNC:2324] | 6629 | -0.052 | -0.3983 | No |
| 8 | ELAVL1 | ELAV like RNA binding protein 1 [Source:HGNC Symbol;Acc:HGNC:3312] | 7426 | -0.113 | -0.4455 | No |
| 9 | MEX3D | mex-3 RNA binding family member D [Source:HGNC Symbol;Acc:HGNC:16734] | 7652 | -0.131 | -0.4461 | No |
| 10 | CPEB3 | cytoplasmic polyadenylation element binding protein 3 [Source:HGNC Symbol;Acc:HGNC:21746] | 8060 | -0.160 | -0.4570 | No |
| 11 | EXOSC8 | exosome component 8 [Source:HGNC Symbol;Acc:HGNC:17035] | 8554 | -0.198 | -0.4697 | No |
| 12 | RBMS3 | RNA binding motif single stranded interacting protein 3 [Source:HGNC Symbol;Acc:HGNC:13427] | 8981 | -0.230 | -0.4730 | No |
| 13 | ZC3H12A | zinc finger CCCH-type containing 12A [Source:HGNC Symbol;Acc:HGNC:26259] | 9030 | -0.234 | -0.4465 | No |
| 14 | ELAVL3 | ELAV like RNA binding protein 3 [Source:HGNC Symbol;Acc:HGNC:3314] | 10273 | -0.348 | -0.4979 | Yes |
| 15 | ELAVL4 | ELAV like RNA binding protein 4 [Source:HGNC Symbol;Acc:HGNC:3315] | 10412 | -0.362 | -0.4619 | Yes |
| 16 | ZFP36L2 | ZFP36 ring finger protein like 2 [Source:HGNC Symbol;Acc:HGNC:1108] | 10509 | -0.374 | -0.4209 | Yes |
| 17 | EXOSC9 | exosome component 9 [Source:HGNC Symbol;Acc:HGNC:9137] | 10606 | -0.384 | -0.3787 | Yes |
| 18 | TIA1 | TIA1 cytotoxic granule associated RNA binding protein [Source:HGNC Symbol;Acc:HGNC:11802] | 10855 | -0.411 | -0.3449 | Yes |
| 19 | TIAL1 | TIA1 cytotoxic granule associated RNA binding protein like 1 [Source:HGNC Symbol;Acc:HGNC:11804] | 11049 | -0.438 | -0.3033 | Yes |
| 20 | KHSRP | KH-type splicing regulatory protein [Source:HGNC Symbol;Acc:HGNC:6316] | 11285 | -0.469 | -0.2609 | Yes |
| 21 | ILF3 | interleukin enhancer binding factor 3 [Source:HGNC Symbol;Acc:HGNC:6038] | 12353 | -0.692 | -0.2544 | Yes |
| 22 | ZFP36 | ZFP36 ring finger protein [Source:HGNC Symbol;Acc:HGNC:12862] | 12846 | -1.052 | -0.1566 | Yes |
| 23 | ZFP36L1 | ZFP36 ring finger protein like 1 [Source:HGNC Symbol;Acc:HGNC:1107] | 12888 | -1.244 | 0.0009 | Yes |
Table: GSEA details [plain text format]

  

Fig 2: GOMF\_MRNA\_3\_UTR\_AU\_RICH\_REGION\_BINDING      
 Blue-Pink O' Gram in the Space of the Analyzed GeneSet

  

Fig 3: GOMF\_MRNA\_3\_UTR\_AU\_RICH\_REGION\_BINDING: Random ES distribution      
 Gene set null distribution of ES for **GOMF\_MRNA\_3\_UTR\_AU\_RICH\_REGION\_BINDING**
